# Protocol for profiling chromatin accessibility in human small-cell lung cancer FFPE-derived samples using Omni-ATAC-seq

**DOI:** 10.1016/j.xpro.2026.104916

**Published:** 2026-10-23

**Authors:** Eralda Salataj, Kenta Kawasaki, Joan Massagué, Charles M. Rudin, Iestyn Whitehouse, Richard Koche, Pierre-Jacques Hamard

**Affiliations:** 1 Center for Epigenetics Research, Memorial Sloan Kettering Cancer Center, New York, NY 10065, USA; 2 Division of Gastroenterology and Hepatology, Department of Internal Medicine, Keio University School of Medicine, Tokyo 160-8582, Japan; 3 Keio Cancer Center, Keio University School of Medicine, Tokyo 160-8582, Japan; 4 Cancer Biology and Genetics Program, Sloan Kettering Institute, Memorial Sloan Kettering Cancer Center, New York, NY 10065, USA; 5 Department of Medicine, Memorial Sloan Kettering Cancer Center, New York, NY 10065, USA; 6 Molecular Biology Program, Sloan Kettering Institute, Memorial Sloan Kettering Cancer Center, New York, NY 10065, USA

**Keywords:** Cancer, Genomics, Molecular Biology

## Abstract

The assay for transposase-accessible chromatin using sequencing (ATAC-seq) on formalin-fixed paraffin-embedded (FFPE) samples maps accessible chromatin in archival tissues, but DNA degradation and crosslinking remain significant challenges when processing these samples. Here, we present a protocol for Omni-ATAC-seq on degraded human small-cell lung cancer (SCLC) FFPE-derived samples. We detail steps for successful nuclei isolation, efficient tagmentation, and optimal library amplification to overcome the low signal-to-noise ratio. This protocol enables high-quality epigenetic profiling, turning archived SCLC samples into valuable resources for studying disease progression.

For complete details on the use and execution of this protocol, please refer to Kawasaki et al.^1^

## Highlights

• Steps for QC of highly degraded FFPE samples
• Steps for rapid nuclei isolation from SCLC FFPE-derived samples
• Instructions for enzymatic and mechanical FFPE tissue dissociation
• Guidance on Tn5 tagmentation and Omni-ATAC-seq library amplification

## Abstract (editor highlights)

Publisher’s note: Undertaking any experimental protocol requires adherence to local institutional guidelines for laboratory safety and ethics.

## Graphical abstract

**Figure:**
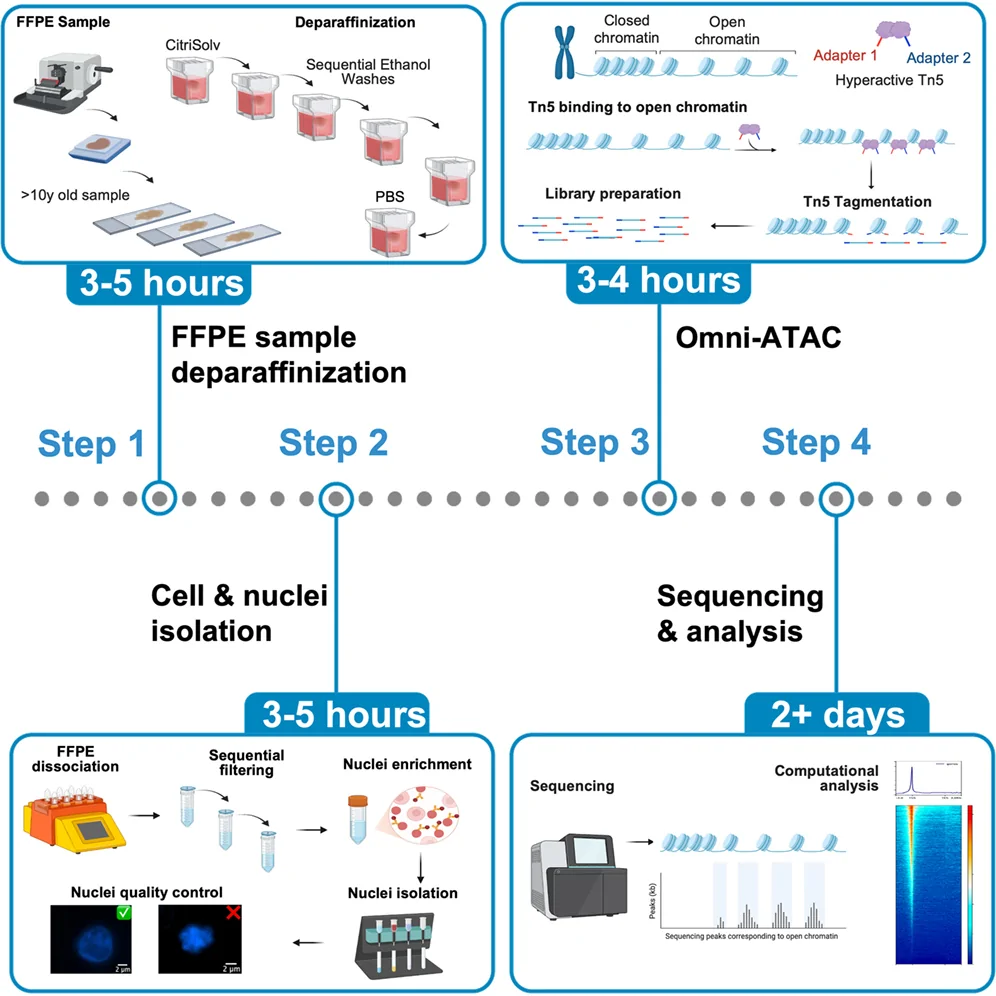

## Before you begin

Formalin-fixed paraffin-embedded (FFPE) tissue represents the standard way for the long-term preservation of patient specimens following surgical resection, or biopsies. Archived FFPE blocks constitute the foundation of biobanking infrastructures worldwide, serving as an invaluable, clinically annotated reservoir for retrospective molecular and epigenetic studies. This preservation process utilizes formaldehyde-induced crosslinking to stabilize structural tissue architecture. However, despite it being highly effective for maintaining morphological features over several decades, this process introduces damage to nucleic acid integrity through chemical crosslinking which leads to DNA degradation.^2^^,^^3^

Over the past two decades, the rapid growth of high-throughput genomic assays has greatly improved our ability to study molecular features at very high resolution.^4^^,^^5^ However, most of these technologies were originally designed/performed either in fresh cells or frozen tissues. To make use of the large number of samples stored in biobanks and pathology archives worldwide, several teams have worked to adapt these methods for formalin-fixed paraffin-embedded (FFPE) tissues. Early studies showed that useful gene expression data could still be obtained from FFPE samples, demonstrating that large-scale molecular profiling was possible in archived clinical material which often contains damaged and fragmented nucleic acids due to the fixation process. Around the same time, the development of ATAC-seq (Assay for Transposase-Accessible Chromatin using sequencing) transformed the study of chromatin accessibility and gene regulation in fresh and frozen tissues.^6^ More recently, improved low-input and FFPE-compatible ATAC-seq methods have extended this technology to archived clinical specimens, allowing researchers to study chromatin accessibility in frozen and FFPE tissues that were previously difficult to analyze.^7^^,^^8^

However, a major barrier to the widespread adoption of current FFPE ATAC-seq methodologies remains their laborious and extensive timelines. Existing published protocols rely on extensive processing, frequently requiring 12–16 hrs incubation to achieve proper chemical decrosslinking and viable nuclei isolation. These prolonged workflows limit sample throughput and increase the risk of further sample degradation. Currently, for highly sensitive FFPE samples like SCLC, there is a critical, unmet need for streamlined protocols that reduce the processing time without compromising library quality or data resolution. To address this limitation, here we present a rapid, optimized workflow that eliminates the requirement for 12–16 hrs processing steps, providing an efficient alternative for scalable retrospective epigenomic discovery.

### Innovation

Our key innovation lies in optimizing the isolation of intact 3D nuclei from small cell lung cancer (SCLC) FFPE-derived samples for subsequent Omni-ATAC analysis, enabling the extraction of high-quality epigenomic information from archived, aged, and highly degraded samples. In order to preserve nuclear integrity and chromatin architecture, we have developed a protocol that minimizes sample exposure to prolonged processing steps, such as overnight incubations, which can further compromise FFPE material. In addition, in this protocol, we have included detailed troubleshooting guidance for profiling highly degraded samples with a DNA Integrity Number (DIN) below 3. Our optimized cell/nuclei isolation strategy incorporates the use of the Miltenyi FFPE Tissue Dissociation Kit in combination with the gentleMACS Dissociator, allowing successful processing from as little as 2–3 sections of FFPE tissue each 10 μm thick and approximately 1 cm^2^ in surface area. Several modifications were introduced to the manufacturer’s protocol, including buffer optimization, sequential filtration steps, adjusted lysis conditions, reduced centrifugation speeds, and complete removal of the epitope retrieval step, as the elevated temperatures associated with this treatment may further damage sensitive FFPE-derived nuclei. This low-input workflow enables efficient and cost-effective ATAC-seq profiling when tissue availability is limited, expanding the use of valuable archival specimens for epigenomic studies and retrospective analyses.

Please refer to the Miltenyi FFPE Tissue Dissociation Kit instructions for additional information and successful use of this protocol.

### Institutional permissions

All experiments conducted as part of this work were reviewed and approved by the institutional committee of Memorial Sloan-Kettering Cancer Center (MSKCC). Human clinical data and archived patient samples were obtained and analyzed under an MSKCC Institutional Review Board (IRB)-approved protocol (14–091). This protocol was conducted in accordance with the ethical guidelines of the Declaration of Helsinki. Prior to downstream molecular processing, all specimens and matching clinical histories were fully anonymized and de-identified to protect patient confidentiality in compliance with MSKCC/HIPAA regulations.

## Key resources table

**Table:** 

REAGENT or RESOURCE	SOURCE	IDENTIFIER
**Chemicals, peptides, and recombinant proteins**		

Ethanol	Fisher Scientific	BP2818-500
CitriSolv	VWR	89426–268
SPRI Magnetic Beads	Beckman Coulter	B23319
DAPI	FisherScientific	P36962
Propidium Iodide (PI)	Denovix	CD-PI-1.5
1× PBS	Fisher Scientific	MT21040CV
NEBNext 2× MasterMix	NEB	M0541L
Qubit™ 1× dsDNA High Sensitivity (HS) and Broad Range (BR) Assay Kits	ThermoFisher Scientific	Q32854 Q32853

**Critical commercial assays**		

FFPE Tissue Dissociation Kit	Miltenyi	130–118–052
Quick-DNA™ FFPE Kit	Zymo Research	D3067
ChIP DNA Clean & Concentrator	Zymo Research	D5205
Anti-Nucleus MicroBeads	Miltenyi	130–132–997
Illumina Tagment DNA Enzyme and Buffer Large Kit	Illumina	20034198

**Biological samples**		

SCLC FFPE-derived clinical samples	MSKCC Pathology Core	N/A

**Software and algorithms**		

ImageJ	Schindelin et al.^9^	https://imagej.net/software/fiji/
IGV	Robinson et al.^10^	https://igv.org/

**Oligonucleotides**		

Nextera Universal i5 primer (AATGATACGGCGACCACCGA GATCTACACTCGTCGGCAGCGTCAGATGTG)	IDT	N/A
Nextera i7 indices (CAAGCAGAAGACGGCATACGAGATXXXXXXXXGT CTCGTGGGCTCGGAGATGTG)	IDT	N/A

**Other**		

PCR thermocycler	Life Technologies	ProFlex PCR System
Centrifuge	ThermoScientific	ST16R
Centrifuge	Eppendorf	5427R
CellDrop Dual Fluorescence + Brightfield Cell Counter	DeNovix	FL-UNLTD
GentleMACS Octo Dissociator with Heaters	Miltenyi	130–096–427
gentleMACS™ Octo Coolers	Miltenyi	130–130–533
Magnetic rack	NEB	S1515S
TapeStation	Agilent	Cat# 4200
HS D5000 Buffer + Ladder (Agilent)	Agilent	Cat# 5067–5589
HS D5000 Tapes (Agilent)	Agilent	Cat# 5067–5588
Microscope Cover Glass	Fisher Scientific	Cat# 50–143–783
Superfrost™ Plus Microscope Slides	Fisher Scientific	22–037–246
Eppendorf™ DNA LoBind™ Tubes	Fisher Scientific	Cat# 13–698–791
0.2 mL PCR Tubes	Eppendorf	Cat# 2231001160
Conical Polypropylene Centrifuge Tubes (15 mL)	Fisher Scientific	Cat# 07–200–886
Falcon™ 50 mL High Clarity Conical Centrifuge Tubes	Fisher Scientific	Cat# 14–432–22
5 mL serological pipette	Fisher Scientific	Cat # 13–675–22
10 mL serological pipette	Fisher Scientific	Cat # 13–675–20
Cell strainer 100μm	Fisher Scientific	22–363–549
Cell strainer 70μm	Miltenyi	130–095–823
Cell strainer 40μm	Fisher Scientific	22–363–547
GentleMACS C tubes	Miltenyi	130–096–334
Eppendorf ThermoMixer C	FisherScientific	2231001005
Qubit 3.0 fluorometer	ThermoFisher Scientific	Cat# Q33216
Qubit Tubes	ThermoFisher Scientific	Cat# Q32856

## Materials and equipment

**Table:** Resuspension Buffer

Reagent	Final concentration	Amount
1M Tris HCl pH 7.5	10mM	500 μL
5M NaCl	10mM	100 μL
1M MgCl2	3mM	150 μL
Sterile H2O	N/A	49.25 mL
**Total**	–	**50 mL**

∗Store at 4^o^C up to 6 months.

**Table:** Lysis Buffer

Reagent	Final concentration	Amount
10% NP40	0.1%	5 μL
10% Tween-20	0.1%	5 μL
5% Digitonin	0.01%	1 μL
ATAC Resuspension Buffer	N/A	489 μL
**Total**	–	**500 μL**

∗ Prepare fresh before each use and store at 4^o^C until needed.

**Table:** Tagmentation buffer

Reagent	Final concentration	Amount
2× Tagment DNA Buffer	1×	25 μL
TDE1 Tagment DNA Enzyme	N/A	2.5 μL
1×PBS	0.01%	16.5 μL
5% Digitonin	0.01%	0.1 μL
10% Tween 20	0.1%	0.5 μL
H2O	N/A	5.4 μL
**Total**	–	**50 μL**

∗ Prepare fresh before each use and store on ice until needed.

### FFPE sample quality control—DNA integrity test

Prior to any downstream FFPE Omni-ATAC-seq assay, we strongly recommend to initially perform a DNA degradation test as a critical quality control step (Figure 1). This assessment provides an estimate of DNA Integrity Number (DIN) and fragmentation levels, enabling samples to be categorized based on their quality. The degradation profile, can be used to guide experimental design and optimize protocol parameters, including input requirements, enzymatic reaction conditions, filtration steps, centrifugation speed and library preparation. For FFPE samples with low DIN values, we recommend following the modified protocol described here, which incorporates specific optimizations and quality control checkpoints throughout the workflow designed to accommodate highly degraded material. Implementing the DNA degradation test quality control, ensures that highly degraded samples are processed under conditions tailored to their level of DNA integrity. DNA from FFPE samples is performed with Quick-DNA™ FFPE Kit following manufacturer’s instructions.

**Figure 1:**
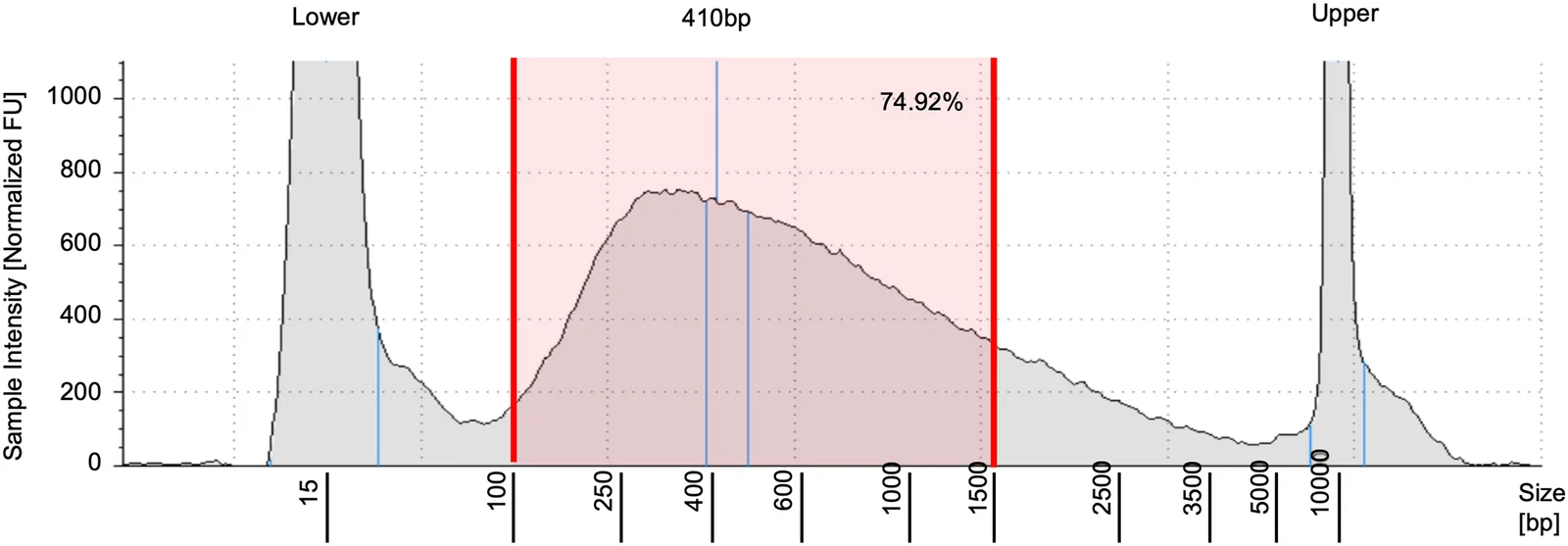
DNA Integrity test on SCLC FFPE-derived samples Electropherogram that displays fluorescence over migration time, allowing accurate determination the fragmentation profile using a D5000 screen tape. The protocol described here can be applied to old archived and highly degraded FFPE samples that show a profile with a distribution of 65% of the material falling within the range of 100–1500 bp (red highlight). The lowermost (15 bp) and the uppermost (10,000 bp) bands represent the lower and upper DNA markers, respectively.

## Step-by-step method details

### FFPE sample deparaffinization

**Timing: 3–5 h**

The goal of this step is to remove the paraffin from the FFPE tissue while minimizing material loss. Upon deparaffinization, the tissue is gradually rehydrated through a series of decreasing ethanol concentrations and finally transferred to PBS, preparing the sample for downstream processing.

1. Prepare 2–3× 10 μm sections from a FFPE block and make sure that each surface is ≥1 cm^2^.

**CRITICAL:** For heterogeneous samples (mix of normal stromal cells and tumor cells), H&E-guided macrodissection can be used to isolate the relevant cell population. Please refer to problem 1 for further troubleshooting.

**CRITICAL:** For this assay, we recommend using 10-μm FFPE tissue sections mounted on positively charged slides). FFPE curls possess a higher risk of sample loss during deparaffinization (washes), whilst FFPE positively charged slides are more stable with minimal material loss. For further details, see problem 2.

2. Transfer slides into Coplin jar.
3. Add fresh CitriSolv to the Coplin jar, making sure that all tissue on the slide is submerged and incubate for 10 min at 22°C–25°C and carefully discard liquid.

***Note:*** CitriSolv is a d-Limonene-based xylene substitute and should be strictly used in glass Coplin jars that are resistant to xylene-based solvents. CitriSolv is toxic, so it is recommended to be handled under a chemical fume hood and wearing the appropriate protection.

4. Repeat two more washes with fresh CitriSolv (3 min each).
5. Add 100% ethanol to the Coplin jar, incubate for 30 s at 22°C–25°C, and carefully discard liquid.

***Note:*** Ethanol serves a dual role here: firstly, it washes away all xylene residuals from the tissue, and secondly, it reintroduces water (through decreasing concentrations) to safely rehydrate the section, for further downstream processing.

6. Repeat step 5 one more time.
7. Add 70% ethanol to the Coplin jar, incubate for 30 s at 22°C–25°C, and carefully discard liquid.
8. Add 50% ethanol to the Coplin jar, incubate for 30 s at 22°C–25°C, and carefully discard liquid.
9. Add 25% ethanol to the Coplin jar, incubate for 30 s at 22°C–25°C, and carefully discard liquid.
10. Add 1× PBS (Ca^2+/^Mg^2+^ free) to the Coplin jar, incubate for no more than 15 min at 22°C–25°C.

### Cell and nuclei isolation

**Timing: 3–5 h**

The goal of this step is to dissociate the deparaffinized FFPE tissue into a single-cell suspension while minimizing cell and nuclear damage. Initially, the tissue is enzymatically dissociated and filtered to remove tissue aggregates and debris, followed by cell lysis to release the nuclei. Nuclei are then isolated and enriched using Anti-Nucleus MicroBeads. The quality and integrity of the isolated nuclei are assessed before proceeding with downstream processing.

11. Carefully scrape the slides (with razor blades).
  a. Collect the material in a 2 mL DNA LoBind Eppendorf tube.
  b. Add 1 mL of non-pre-heated Buffer U from the Miltenyi FFPE Tissue Dissociation Kit.
  c. Incubate for 30 s at 22°C–25°C, and aspirate liquid.

***Note:*** At this point the material is very sticky and will stick to your tip so carefully try to remove as much supernatant as possible without compromising your deparaffinized material.

12. Add 1 mL of Buffer V from the Miltenyi FFPE Tissue Dissociation Kit, incubate for 30 s at 22°C–25°C, and aspirate the liquid.

***Note:*** At this point, the material remains sticky and may adhere to the pipette tip. Carefully remove as much supernatant as possible without losing the deparaffinized material.

13. Prepare the enzymatic cocktail following the exact composition of the dissociation mix by adding 2.3 mL Buffer V, 100 μL of dissolved Enzyme D, 100 μL of dissolved Enzyme G, and 2.5 μL of Reagent A per sample. Add a small volume of the enzymatic cocktail to the DNA LoBind tube to rinse and recover the FFPE material, then transfer the recovered material to a gentleMACS C Tube. Add the remaining enzymatic cocktail to the same gentleMACS C Tube and tightly close it.

***Note:*** For the above enzymatic reaction, the total tissue input should not exceed 50 μm, as per manufacturer’s instructions.

***Note:*** Close C Tube tightly beyond the first resistance.

14. Run the gentleMACS Program 37C_FFPE_1 on the gentleMACS Octo Dissociator with Heaters (47 min).
  ***Note:*** Before starting, make sure that the gentleMACS Program 37C_FFPE_1 includes these steps. All the below steps (incubation time and rotation directions) are included in the standard gentleMACs protocol:
  a. Temp ON.
  b. Spin −20 rpm, 5′ 0″ *(counterclockwise)*.
  c. loop 3×.
  d. spin 20 rpm, 14′ 0″.
  e. spin 1700 rpm, 7″.
  f. spin 1700 rpm, 1″.
  g. spin −1700 rpm, 2″ *(counterclockwise)*.
  h. spin 1700 rpm, 1″.
  i. spin 1700 rpm, 4″.
  j. end loop.
  k. end.
15. After termination of the program, detach C Tube from the gentleMACS Octo Dissociator with Heaters and place the C Tube on ice.

**CRITICAL:** At this point if a visible pellet is not recovered, please see problem 3 for further troubleshooting.

**CRITICAL:** Please make sure that there are no aggregates visible. If aggregates are still present then, please see problem 4 for further troubleshooting.

16. Apply the cell suspension to a Pre-Separation Filter (100 μm) placed on a 15 mL tube on ice.
17. Wash the filter with 1 mL ice-cold Buffer W.

***Note:*** Alternatively, for maximum cell recovery, rinse the used C Tube with 1 mL ice-cold Buffer W before adding it to the filter.

18. Discard the filter and centrifuge cell suspension at 1000 × *g* at 4°C for 5 min. Aspirate supernatant completely.
19. Add 5 mL of buffer W and apply the cell suspension to a Pre-Separation Filter (70 μm) placed on a 15 mL tube on ice.
20. Discard the filter and centrifuge cell suspension at 1000 × *g* at 4°C for 5 min. Aspirate supernatant completely.
21. Add 5 mL of buffer W and apply the cell suspension to a Pre-Separation Filter (40 μm) placed on a 15 mL tube on ice.
22. Discard the filter and centrifuge cell suspension at 1000 × *g* at 4°C for 5 min. Aspirate supernatant completely.
23. Resuspend cells in 1 mL of cold 1×PBS and determine cell quality using either DAPI or PI staining. (Figure 2A).

**Figure 2:**
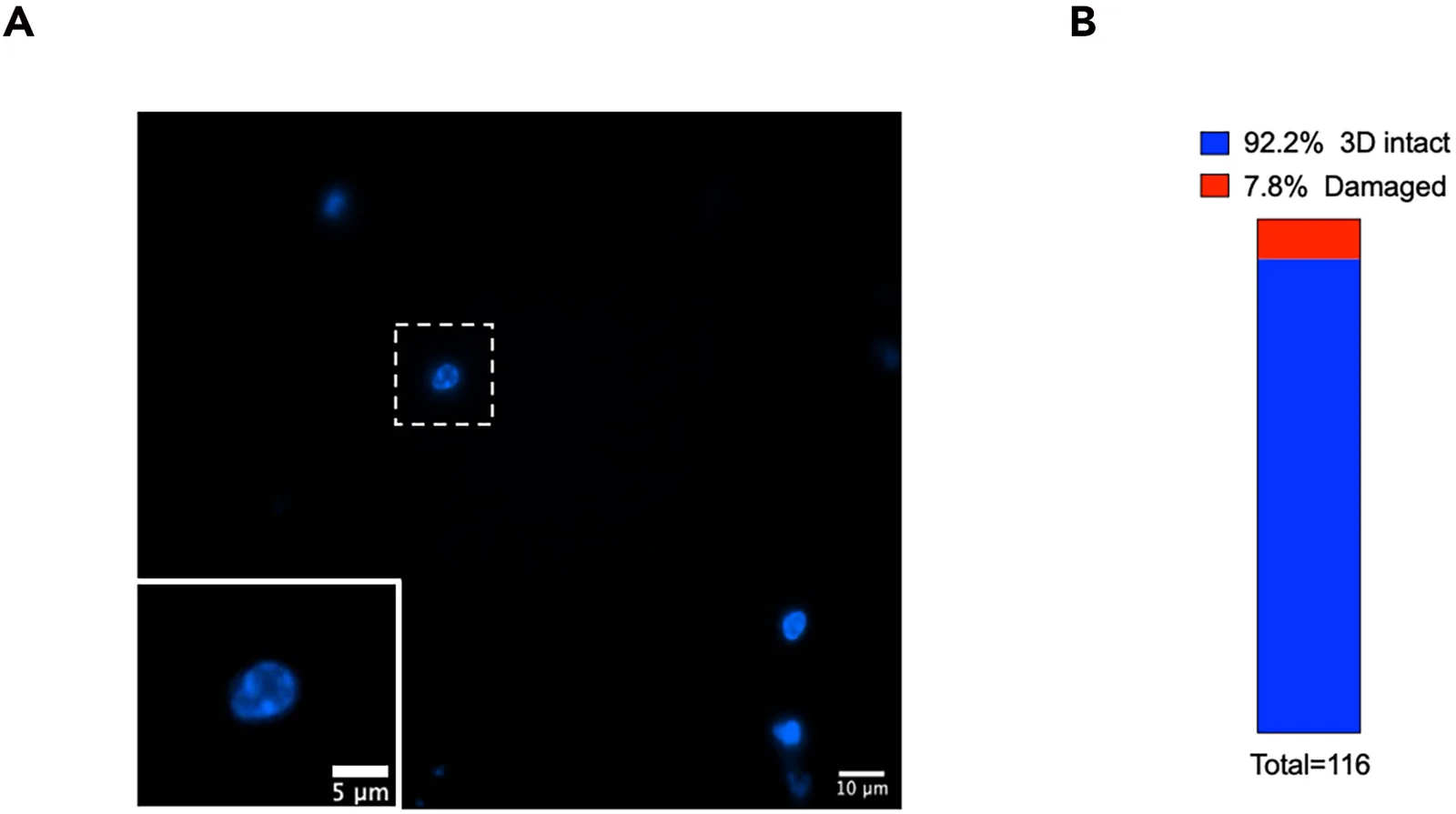
FFPE Cell isolation (A) Representative single z-stack images of SCLC cells isolated from FFPE tissue samples (left). Following isolation, cells were cytospun onto coverslips and stained with DAPI. Insets highlight representative healthy nuclei with preserved three-dimensional (3D) architecture, characterized by smooth and intact nuclear boundaries as visualized by DAPI staining, in SCLC FFPE-derived sample. Scale bars, 5 μm (inset) and 10 μm. (B) Stacked bar chart showing the percentage of nuclei displaying 3D intact shape vs nuclei that were identified as showing a disrupted 3D architecture. Number of nuclei assessed, *n* = 116.

**CRITICAL:** If single cells comprise <70% of the material, DAPI-based sorting is strongly recommended. In our lab we identify the 3D-intact single cells, based on smooth perinuclear edges, as defined by DAPI staining, and by confirming that they were not part of cell aggregates. Each image usually consists of approximately 25–40 optical sections and is acquired by an epifluorescence microscope on a 100× objective. Maximum projections of the images are generated using ImageJ analysis software, cells are counted manually usually by two independent investigators. Do not proceed with nuclei isolation if single cells are <70%. Please refer to problem 5 for further details.

**CRITICAL:** If the majority of cells do not display a round morphology and show impaired integrity, the cell isolation needs to be further troubleshooted. If upon isolation more than 10% of cells show disrupted 3D nuclear architecture (Figure 2B), we recommend further troubleshooting. For details, see problem 6.

24. Transfer cells in Eppendorf tube and pellet the cells at 1000 × *g* at 4°C for 5 min.
25. Aspirate all supernatant, carefully avoiding visible cell pellet, using two pipetting steps (aspirate down to 100 μL with a P1000 pipette and remove final 100 μL with a p200 pipette).
26. Add 50 μl cold ATAC-lysis buffer and pipette up and down 3 times.
27. Incubate on ice for 8–10 min.

**CRITICAL:** Depending on tissue type (e.g: FFPE sarcoma-derived samples), additional 2–3-min incubation on ice after dissociation might be needed to optimize cell lysis. Over-lysis should be avoided as it could lead to nuclei aggregation and nuclei damage or nuclei explosion. For further details, see problem 7.

28. Pellet nuclei at 300 × *g* for 10 min at 4°C.
29. Resuspend nuclei pellet with ice-cold resuspension buffer by slowly and gently pipetting the sample up and down for 10 times.
30. Collect nuclei suspension and determine nucleus number.
31. Centrifuge nuclear suspension at 300 × *g* for 5 min. Pipette off supernatant completely.
32. Resuspend nuclear pellet in 450 μL of nuclei separation buffer per 10^6^ total nuclei.
33. Add 50 μL of Anti-Nucleus MicroBeads per 10^6^ total nuclei.
34. Mix well and incubate for 15 min in the refrigerator (2°C–8°C).
35. Add 2 mL nuclei separation buffer (independent from nucleus sample input) and proceed to magnetic separation with LS columns.
36. Place column in the magnetic field of a suitable MACS Separator.
37. Prepare two columns by rinsing with 3 mL of nuclei separation buffer.
38. Apply nucleus suspension onto the column. Collect flow-through containing debris.

***Note:*** Since the volume of the buffer should be kept low, add the nucleus suspension directly to the center of the column reservoir.

39. Wash column two times with 1 mL of nuclei separation buffer. Collect debris that passes through and combine with the flow-through from step 38.

***Note:*** Perform washing steps by adding buffer aliquots as soon as the column reservoir is empty.

40. Remove column from the separator and place it on a suitable collection tube.
41. Pipette the appropriate amount of nuclei separation buffer onto the column depending on nucleus sample input. Immediately flush out the magnetically labeled nuclei by firmly pushing the plunger into the column.
42. Centrifuge at ∼300 × *g* for 3 min and resuspend the cell pellet in the supernatant.
43. Count nuclei with a PI or DAPI staining and identify how many nuclei present an intact 3D structure.

**CRITICAL:** If there are less than 100,000 nuclei per condition then nuclei isolation needs further troubleshooting. Please see problem 8:

44. Use precisely 100,000 nuclei/condition and keep the nuclei on ice in 1×PBS until the next steps.

### Omni-ATAC

**Timing: 3–4 h**

The goal of this step is to identify and sequence regions of accessible chromatin from the isolated FFPE nuclei. Initially, nuclei are subjected to Tn5-mediated transposition, which fragments accessible DNA. Following transposition, DNA is then purified and sequentially PCR-amplified to generate sufficient material for sequencing. The amplified libraries are then purified, assessed for quality and fragment size distribution, and prepared for short-read sequencing.

45. Resuspend nuclei pellet in 50 μL of transposition mixture by gently pipetting up and down 8–10 times.
46. Incubate reaction at 37°C for exactly 35 min in a thermomixer with 1400 RPM mixing.

***Note:*** When starting with 100,000 nuclei, we recommend a 35-min tagmentation incubation. In our experience, shorter incubation times can result in incompletely or partially tagmented samples.

47. Place on ice directly following cycle and proceed with DNA isolation/purification by using Zymo PCR Purification Kit.
48. Add 5μL 3M Sodium Acetate and 250 μL Binding Buffer to each collection tube (Do not quick spin after this step).
49. Place a Zymo-Spin™ column in a 2 mL collection tube.
50. Transfer all the sample to a Zymo-Spin™ column and centrifuge for 1 min at 12000 × *g*.
51. Discard flow-through. Place the Zymo-Spin™ column back into the same collection tube.
52. Add 750 μL Buffer PE to the Zymo-Spin™ column and centrifuge for 1 min @ 12000 g.
53. Discard the flow-through and place the column back in the same collection tube. Centrifuge the Zymo-Spin™ column for an additional 1 min at 12000 × *g*.
54. Place the Zymo-Spin™ column in a clean 1.5 mL DNA low binding tube.
55. Add 20 μL Buffer EB to the center of the membrane incubate for 3 min at 22–25°C and then centrifuge at 24000 × *g* for 1 min.

***Note:*** After purification, DNA can be stored at −20°C (Pause point).

56. PCR amplification:

**Table:** PCR reaction master mix

Reagent	Amount
DNA Tagmented	20 μL
NEBNext 2× MasterMix	25 μL
Primer 1 (25 μM) i5	2.5 μL
Primer 2 (25 μM) i7	2.5 μL

57. Amplify for 10 cycles using NEBNext 2× MasterMix.

***Note:*** In our hands, 10 cycles are the adequate number for amplification. However, in some cases, determining the required number of cycles by qPCR can be helpful, as described in Buenrostro et al, 2013.

**Table:** PCR cycling conditions

Steps	Temperature	Time	Cycles
Initial extension/gap-fill	72°C	5 min	1
Initial denaturation	98°C	30 sec	1
Denaturation	98°C	10 sec	10 cycles
Annealing & extension	63°C	30 sec	
Final extension	72°C	1 min	1
Hold	8°C	forever

58. When PCR reaction is completed, remove samples and transfer them on ice and proceed with DNA purification.
59. Add 25 μL (0.5×) SPRI beads to each sample and mix thoroughly by pipetting up and down 10 times.
60. Incubate at 22°C–25°C for 10 min.
61. Place samples on a magnetic rack for 2 min until the solution clears.
62. Carefully transfer the supernatant to a new PCR tube.
63. Add 65 μL (1.3×) SPRI beads to the recovered supernatant and mix by pipetting up and down 10 times.
64. Incubate at 22°C–25°C for 10 min.
65. Place samples on a magnetic rack for 2 min until fully cleared.
66. Remove and discard the supernatant without disturbing the bead pellet.
67. Wash beads twice with 200 μL freshly prepared 80% ethanol.

***Note:*** Do not allow ethanol to sit on the beads for extended periods; add and remove immediately during each wash.

68. Air-dry beads at 22°C–25°C for 5–8 min until small surface cracks become visible (do not over-dry).
69. Remove tubes from the magnetic rack.
70. Add 20 μL elution buffer (EB) to resuspend the beads and elute DNA.
71. Mix by pipetting up and down 10 times.
72. Place samples on a magnetic rack for 2 min.
73. Transfer the supernatant to a new tube.
74. Tapestation QC (Figure 3).

**Figure 3:**
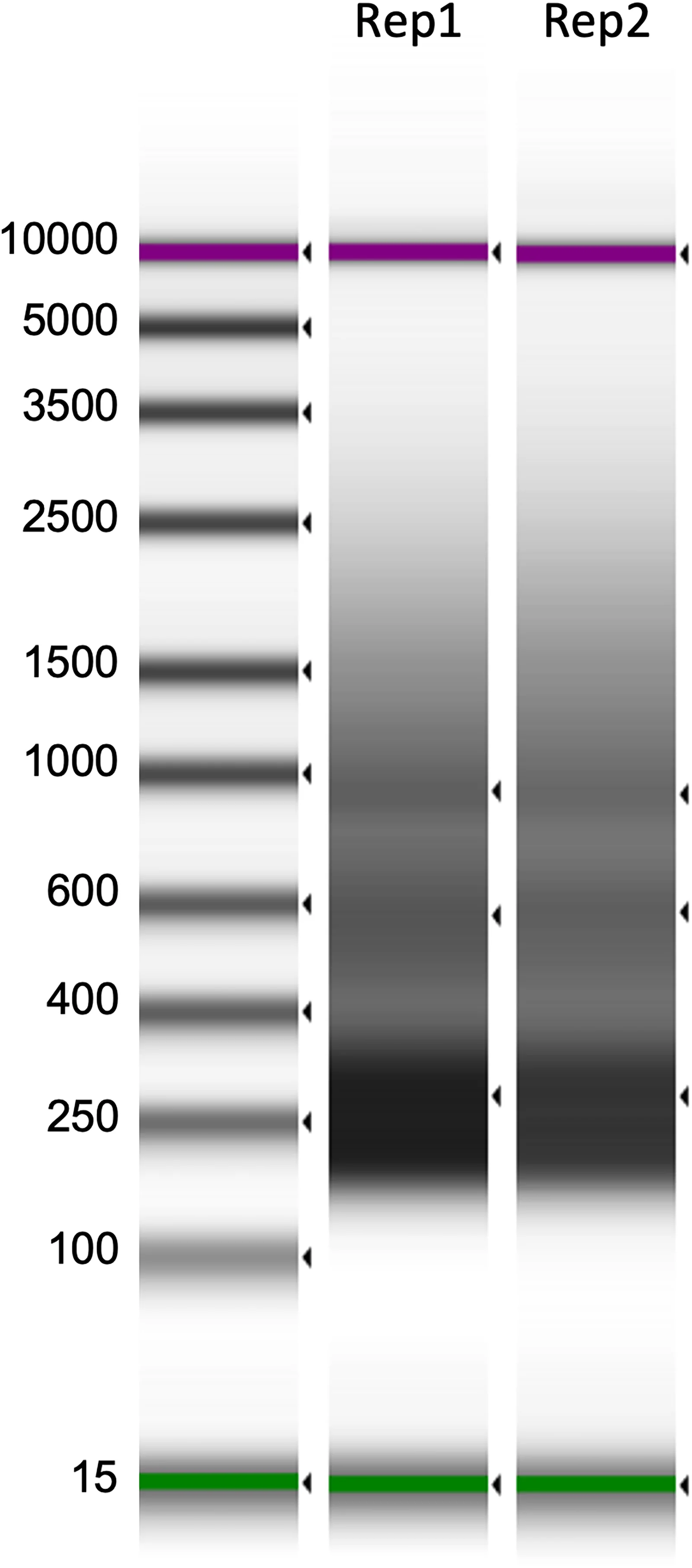
FFPE Omni-ATAC sequencing library Gel image of an optimal FFPE Omni-ATAC-seq library generated from SCLC FFPE-derived cells, showing the expected fragment size distribution with distinct mono-, di-, and tri-nucleosomal bands. This pattern is indicative of preserved chromatin structure and successful library preparation for chromatin accessibility profiling. The lowermost (15 bp) and the uppermost (10,000 bp) bands represent the lower and upper DNA markers, respectively.
75. Purified samples are ready for short-read sequencing.

***Note:*** For FFPE Omni-ATAC-seq, we recommend a sequencing depth of 75–85M PE100 reads per library.

## Expected outcomes

Successful execution of this protocol yields high-quality, complex sequencing libraries that faithfully recapitulate chromatin accessibility landscapes from archived clinical specimens. Initial quality control assessment using an Agilent Bioanalyzer or TapeStation should reveal a characteristic nucleosomal laddering pattern, resolved into distinct peaks representing mono-di-tri nucleosomal pattern (Figure 3). Following paired-end high-throughput sequencing, libraries are expected to demonstrate robust alignment performance, typically yielding unique genomic mapping rates exceeding 70% with managed duplication levels compatible with recovering sufficient non-redundant fragments after filtering. At the chromatin landscape level, high-quality datasets display sharp enrichment of accessibility signals localized at Transcription Start Sites (TSS), consistently achieving a TSS enrichment score (Figure 4A). Despite the FFPE starting material showing high degradation levels, regions with high chromatin accessibility should display low background signal (Figure 4B).

**Figure 4:**
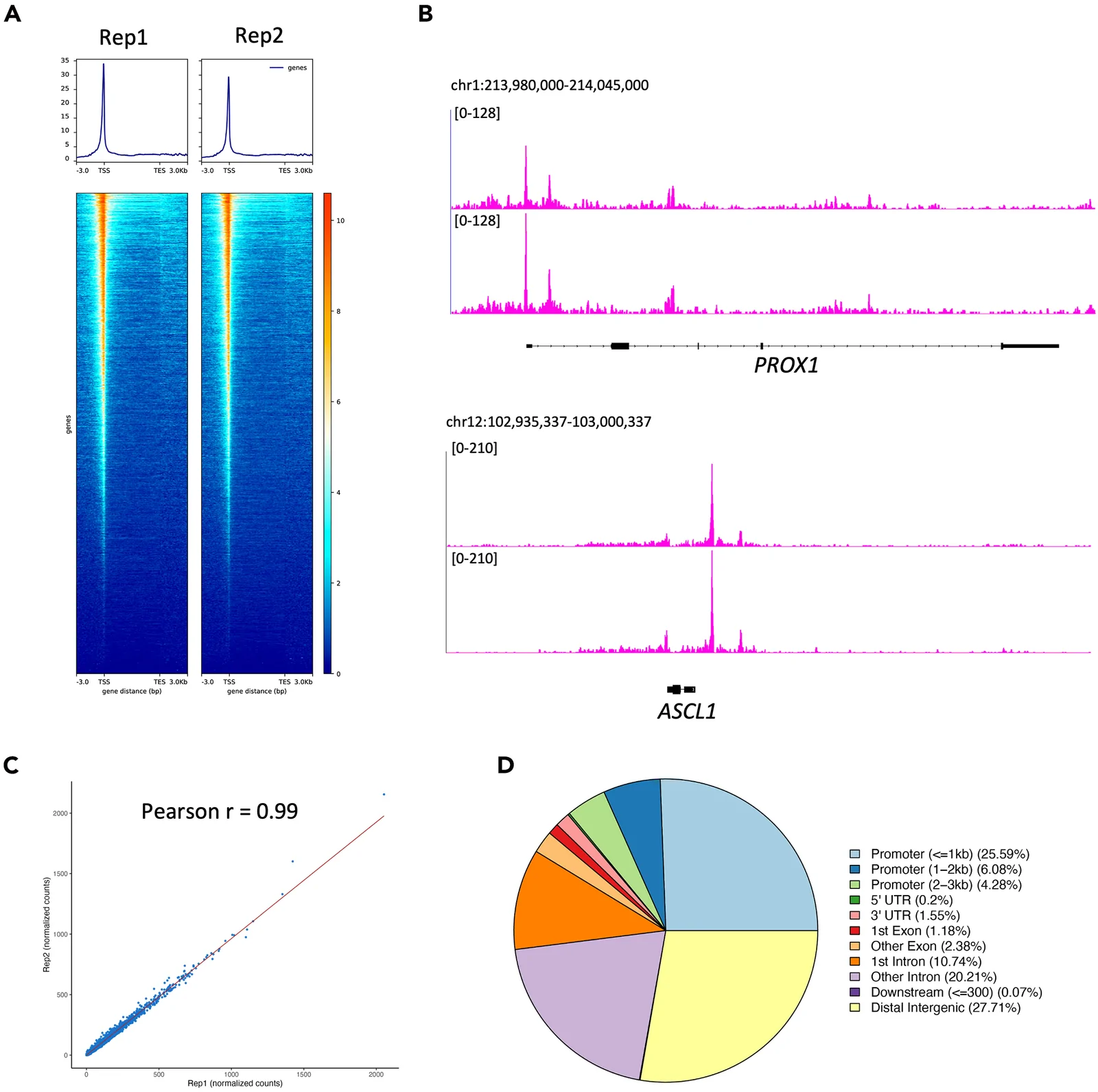
FFPE Omni-ATAC data (A) Heatmap profile of open chromatin regions peaks relative to the transcription start sites (TSS) considering a ±3.5 kb region. Red color intensity reflects the level of peak enrichment. (B) Representative IGV tracks at the *ASCL1* and *PROX1* gene loci in one SCLC FFPE sample. Two technical replicates are shown. (C) Pearson correlation between technical replicates. (D) Distribution of accessible regions.

For this FFPE-Omni-ATAC protocol, a total of 75M to 85M reads were sequenced per sample on an Illumina NovaSeq 6000 flow cell in PE100 mode. The final libraries had an alignment rate >94%, a fraction of reads in peaks (FRiP) score of > 0.44 (ENCODE acceptable threshold is 0.2 and ideal > 0.3), and TSS enrichment > 5.3 (ENCODE acceptable threshold is 5 and ideal > 7). Pearson correlation between technical replicates was 0.99 (Figure 4C). Peaks per sample ranged from 85k to 95k. To better control for additional variability that may arise from FFPE tissues, the data were normalized to accessibility at housekeeping gene promoters in DESeq2 followed by applying those scaling factors to all peaks genome-wide. Normalizing the data to genomic regions where more consistent chromatin accessibility is expected across a range of cell types allows scaling factors to be calculated based on robust signal and avoids the variability that is sometimes introduced by pure sequencing depth normalization or all-peaks normalization.

## Limitations

While this optimized FFPE Omni-ATAC-seq workflow significantly streamlines chromatin accessibility profiling in archived specimens, several technical limitations due to formalin-fixed paraffin-embedded (FFPE) material must be considered. Formaldehyde-induced cross-linking and long-term ambient storage introduce degradation, resulting in severe nucleic acid fragmentation and a reduced signal-to-noise ratio compared to fresh or frozen samples. Furthermore, delays between tissue collection, crosslinking, paraffinization, and extended ischemia time, will cause degradation of endogenous DNA and RNA but also favor the proliferation of bacteria such as *Escherichia coli*, ultimately contributing to increased background noise, contamination of sequencing libraries, and alignment-related artifacts during downstream analysis. ATAC-seq libraries of degraded FFPE samples often exhibit low complexity and elevated PCR duplication rates. Furthermore, because weaker or transient distal regulatory elements—such as cell-type-specific enhancers—can be obscured by background noise in highly degraded blocks, this method is most robust for mapping highly accessible promoter regions, but is also capable of enriching for putative enhancer regions (Figure 4D). Finally, achieving reproducible data depends heavily on accounting for tissue heterogeneity; specimens with dense stromal infiltration or mixed cellular compartments require precise microdissection to prevent the dilution of the target epigenetic signal.

## Troubleshooting

### Problem 1

Heterogenous sample (mixed cancerous and stromal cells, step 1).

### Potential solution

For homogenous tissues we recommend using unstained sections, however for heterogeneous samples we recommend using a matching, stained hematoxylin and eosin (H&E) slide as a guide control, to locate the cancerous regions on adjacent unstained slides. This H&E reference slide, ensures precise selection of region of interest, minimizes sampling bias and enhances assay reproducibility for downstream analysis. Although for tissues with distinct tumor cells, H&E-guided macro-dissection provides a cost-effective enrichment, however because its resolution is limited, it cannot separate stromal infiltration.

### Problem 2

Material detaching upon deparaffinization/rehydration (step 1).

### Potential solution

Robust ATAC-seq profiling depends on sufficient FFPE starting material, prioritizing large surgical resections over needle-core biopsies. For this protocol we strongly recommend using a minimum 1 cm^2^ input of well-preserved tissue to ensure adequate recovery of intact nuclei, utilizing 10 μm-thick sections to optimize structural stability while preserving nuclear integrity. We recommend that samples are mounted on positively charged slides to exploit electrostatic attraction and prevent tissue detachment. Although a standard 10 μm section yields up to 1×10^6^ cells, output declines sharply in necrotic/pre-apoptotic samples. For archived slides (>10 years) stored at −20^o^C, and in order to prevent detachment, slides must equilibrate for 20–30 min in 22°C–25°C while preventing ambient humidity from condensation. During deparaffinization with CitriSolv and subsequent ethanol rehydration, replace aggressive shaking with passive dipping to minimize mechanical stress on the tissue. CitriSolv and ethanol should be added gently against the slide perimeter rather than directly onto the section in order to prevent detachment.

### Problem 3

Not enough material obtained after GentleMACS dissociation (step 15).

### Potential solution

If after the GentleMACS process no material is observed in the C tube that means that dissociation is problematic. A simple explanation could be that the starting material was not enough. ATAC-seq profiling from FFPE samples is highly dependent on adequate starting input. For this protocol we strictly recommend using large surgical resections over the needle-core biopsies. Biopsies usually have limited material which is not sufficient for this multi-step protocol. We strongly suggest using a minimum of ∼1 cm^2^ material of well-preserved FFPE tissue to ensure sufficient recovery of intact cells/nuclei for downstream chromatin accessibility profiling. While a single 10 μm section of FFPE-derived SCLC samples can yield up to ∼1×10^6^ cells under optimal conditions, the effective nuclei recovery may be substantially reduced due to the multi-step process. On top of it, in tissues with preapoptotic cells or poor fixation or overfixation will also yield a lower number of cells. To maximize the single-cell yield and library complexity, it is recommended to pool 3–5 serial sections of 10 μm thickness. Please be aware that more than 50 μm of FFPE material will require adjustment of the step 13 of this protocol (further instructions and details are provided on the Miltenyi FFPE Tissue Dissociation Kit). For heterogeneous samples, a matched hematoxylin and eosin (H&E)–stained reference section should be used to guide macro-dissection of viable tumor regions. This step enables enrichment of highly cellular areas while excluding apoptotic or necrotic regions that contribute to background noise and reduce effective chromatin signal. Based on our experience working with highly sensitive FFPE-derived SCLC samples and other tissues, another factor that would strongly help, is reducing the mechanical force: at step 14e) until step 14i) the 1700 rpm should be replaced with a lower speed of 1200 rpm. This way the sensitive tissue can be dissociated in a gentler way, single cells will be isolated, and no explosion of cells will be observed. For highly sensitive samples we also recommend minimizing the filtration step (avoiding the 70 μm filtration step.

### Problem 4

Aggregates visible upon cell dissociation (step 15).

### Potential solution

If after the GentleMACS dissociation big chunks of material are observed in the C tube that’s an indication of partial enzymatic digestion and insufficient disruption of FFPE tissue, resulting in partial cell release and reduced single-cell suspension quality. To improve dissociation efficiency, a few key parameters can be optimized: enzymatic digestion time, enzyme concentration and increased mechanical dissociation. In our experience, particularly for sensitive small-cell lung cancer (SCLC) samples, increasing the number of dissociation cycles (Step 14 iii)) from 3 to 4–5 cycles substantially improves tissue breakdown and increases total cell yield. Alternatively, enzymatic activity can be increased by raising the concentration of dissociation enzymes, specifically Enzyme D and Enzyme G (following manufacturer’s instructions), to improve dissociation efficiency. However, it is critical that only one parameter is modified at a time (either digestion time or enzyme concentration), as simultaneous adjustment of both conditions may result in overdigestion, leading to compromised cell integrity, an increased number of cellular debris affecting the material and its suitability for downstream chromatin accessibility profiling. Overall, we recommend that a few troubleshooting rounds of dissociation conditions before processing the final samples, in order to balance efficient tissue breakdown, ensure preservation of 3D cellular and nuclear integrity, particularly in FFPE-derived SCLC clinical specimens.

### Problem 5

Obtaining cell aggregates upon filtration and partial single cell suspension (step 23).

### Potential solution

One potential strategy to improve enrichment for intact single cells is the introduction of sequential rounds of pre-separation filtration and/or fluorescence-activated cell sorting (FACS). If, after step 23 of this protocol, sufficient material remains available to proceed (we recommend that this approach should not be considered for samples containing fewer than 1000000 cells), an additional filtration step using a 40 μm pre-separation filter may help reduce the presence of large cellular aggregates and improve the overall quality of the single-cell suspension. However, one should keep in mind that each additional filtration step usually leads to sample loss, particularly when processing fragile or partially degraded material that contains necrotic/pre-apoptotic cells. For this reason, we recommend that implementation of the additional filtration step should be performed only in high-input samples with sufficient cellular recovery. Performing an extra filtration step in low-yield specimens may substantially decrease total cell numbers and will affect nuclei isolation efficiency and further downstream chromatin accessibility profiling. If this additional filtration step does not help to get single cell suspension, then Fluorescence-Activated Cell Sorting (FACS) the cells would be another possible strategy to enrich for intact single cells. DAPI exclusion during FACS can be used to eliminate damaged or permeabilized cells, as well as highly granular debris but also aggregated populations. However, it should be taken into consideration that the introduction of this additional sorting step will increase the overall processing time required to complete this protocol.

### Problem 6

Cells appear exploded without smooth edges and perinuclear leakage (step 23).

### Potential solution

This phenotype is typically indicative of overly harsh mechanical and/or centrifugal stress during the dissociation and wash steps, resulting in compromised membrane integrity and partial nuclear leakage. Given that cells that have been isolated from the FFPE samples are particularly susceptible to mechanical disruption, the dissociation and handling steps can be made more gentle throughout the workflow. Centrifugation force during all wash and pelleting steps can be reduced (e.g., steps 18, 20, 22 and 24 should be ∼300 × *g*), as excessive centrifugal force may compromise cell integrity and result in cell disruption. In addition, gentle pipetting should be performed throughout the protocol, and the introduction of air bubbles during sample handling should be avoided, as this may further damage fragile cells. Another approach to reduce the presence of exploded cells is to enrich for intact 3D cells using a fluorescence-activated cell sorting (FACS) step based on DAPI staining. Please see problem 5 for further troubleshooting.

### Problem 7

Nuclei exploded without smooth edges and perinuclear leakage (step 27).

### Potential solution

If the nuclei are observed to appear “exploded,” with ragged borders and loss of perinuclear integrity, this indicates that the nuclei isolation procedure has been performed under overly harsh conditions, resulting in compromised nuclear structure. Given that the cells were initially observed to display good morphology prior to nuclei isolation, this suggests that the disrupted 3D nuclear phenotype is likely introduced during the lysis and/or nuclei extraction steps rather than reflecting the starting material quality. In our hands this phenotype is usually caused by over-lysis prior to nuclei isolation, where excessive exposure to permeabilization buffer or increased detergent concentration leads to disruption of the nuclear membrane and loss of 3D structural integrity. Therefore, lysis conditions (incubation time and detergent concentration) should be systematically titrated and optimized prior to the final experiment in order to define appropriate nuclei isolation parameters for SCLC FFPE samples. In addition, handling during the lysis step should be performed with care to minimize additional mechanical stress, as this may further exacerbate nuclear damage. Lastly, the centrifugation step should also be performed carefully and never use more than 300 × *g* for pelleting FFPE-derived nuclei.

### Problem 8

Low yield of nuclei isolated-not enough material to proceed with tagmentation (step 43).

### Potential solution

Low nuclei yield is commonly observed in highly sensitive and degraded samples where the nuclear membrane usually explodes after mechanical stress. Overall, we recommend that one of the primary key considerations if this problem appears quite often, would be to increase the amount of starting material, in order to compensate for inevitable losses occurring during mechanical and chemical dissociation steps for fragile samples. Another factor that affects the low nuclear yield is the over-lysis during nuclei isolation process (step 26 of this protocol). Extended exposure to lysis buffer or prolonged incubation time, or overly vigorous pipetting can compromise nuclear membrane integrity and result in nuclei explosion and complete lysis of the material. Therefore, we highly recommend that lysis conditions should be carefully optimized based on samples needs. Also, samples handling should be done as gentle as possible in order to protect the 3D nuclear integrity. Since this protocol includes multiple steps that may increase mechanical stress, special attention should be given to minimizing filtration steps in highly sensitive samples. For example, if the sample exhibits good dissociation and sufficient yield after the 70 μm filtration step, it is recommended to proceed directly to nuclei isolation and avoid the additional 40 μm filtration. At this stage, the 40 μm filter may significantly reduce nuclei recovery, particularly in low-input or fragile specimens, as nuclei can be retained on the filter matrix or lost during transfer. When filtration is required for removal of large debris, it should be performed with caution and only when sufficient starting material is available. Overall, we suggest that detailed optimization of a) input material, b) stringent control of lysis conditions, and c) minimization of unnecessary filtration steps are critical parameters for maximizing the nuclei yield and ensuring robust performance and reproducibility for the downstream analysis.

## Resource availability

### Lead contact

Further information and requests for resources and reagents should be directed to and will be fulfilled by the lead contact, Pierre-Jacques Hamard (hamardp@mskcc.org).

### Technical contact

Technical questions on the protocol performance and details should be directed to and will be answered by the technical contact, Eralda Salataj (salatae1@mskcc.org).

### Materials availability

This study did not generate new unique reagents.

### Data and code availability

This study did not generate or analyze new datasets or code.

## Acknowledgments

We acknowledge the use of the Integrated Genomics Operation Core, funded by the NCI Cancer Center Support Grant (CCSG, P30-CA008748), Cycle for Survival, and the Marie-Josée and Henry R. Kravis Center for Molecular Oncology. This study was supported by National Cancer Institute grant R35-CA252978 (J.M.), the Druckenmiller Center for Lung Cancer Research (K.K. and C.M.R.), and the Alan and Sandra Gerry Metastasis and Tumor Ecosystems Center (J.M.). The graphical abstract was created in Biorender https://www.biorender.com.

## Author contributions

Conceptualization, E.S. and P.-J.H.; methodology, E.S., R.K., and P.-J.H.; clinical samples, C.M.R.; investigation, all authors; formal analysis, all authors; writing – original draft, E.S.; writing – review and editing, E.S., R.K., and P.-J.H.; visualization, E.S., R.K., and P.-J.H.

## Declaration of interests

R.K. is a co-founder of and consultant for Econic Biosciences. J.M. holds company stock from Scholar Rock. C.M.R. has consulted on oncology drug development with AbbVie, Amgen, AstraZeneca, Boehringer Ingelheim, Daiichi Sankyo, Genentech/Roche, Jazz, and Merck and serves on the scientific advisory boards of Auron, Bridge Medicines, DISCO, Earli, and Harpoon Therapeutics. C.M.R. has received licensing fees and royalties based on DLL3 antibody-based therapeutics.
